# Supplementary material for: Identification of Genomic Regions Implicated in Susceptibility to Schistosoma mansoni Infection in a Murine Backcross Genetic Model
Source: Int J Mol Sci. 2023 Sep 30;24(19):14768. doi: 10.3390/ijms241914768 (PMC10573152; doi:10.3390/ijms241914768)
Supplement: Supplementary file 1 [file ijms-24-14768-s001.zip › ijms-2612175 Supplementary material.pdf]

International Journal of
Molecular Sciences

MDPI

Article

Identification of Genomic Regions Implicated in Susceptibility to *Schistosoma mansoni* Infection in a Murine Backcross Genetic Model

Juan Hernández-Goenaga [1], Julio López-Abán [1,*], Adrián Blanco-Gómez [2,3], Belén Vicente [1], Francisco Javier Burguillo [4], Jesús Pérez-Losada [2,3] and Antonio Muro [1,*]

[1] Grupo de Enfermedades Infecciosas y Tropicales (e-INTRO), IBSAL-CIETUS (Instituto de Investigación Biomédica de Salamanca, Centro de Investigación de Enfermedades Tropica-les de la Universidad de Salamanca), Facultad de Farmacia, Universidad de Salamanca, Ldo. Méndez Nieto s/n, 37007 Salamanca, Spain; juhego@usal.es (J.H.-G.); belvi25@usal.es (B.V.)

[2] Instituto de Investigación Biomédica de Salamanca (IBSAL), Complejo Asistencial Universitario de Salamanca, Hospital Virgen de la Vega, 37007 Salamanca, Spain; adrianblanco@usal.es (A.B.-G.); jperezlosada@usal.es (J.P.-L.)

[3] Instituto de Biología Molecular del Cáncer (IBMCC), Centro de Investigación del Cáncer (CIC)—CSIC, Laboratory 20 37007 Salamanca, Spain

[4] Departamento de Química-Física, Facultad de Farmacia, Universidad de Salamanca, C/Donantes de Sangre s/n. Campus Unamuno, 37007 Salamanca, Spain

* Correspondence: jlaban@usal.es (J.L.-A.); ama@usal.es (A.M.); Tel.: +34-923294535 (J.L.-A.)

Citation: Hernández-Goenaga, J.; López-Abán, J.; Blanco-Gómez, A.; Vicente, B.; Burguillo, F.J.; Pérez-Losada, J.; Muro, A. Identification of Genomic Regions Implicated in Susceptibility to *Schistosoma mansoni* Infection in a Murine Backcross Genetic Model. *Int. J. Mol. Sci.* **2023**, *24*, 14768. https://doi.org/10.3390/ijms241914768

Academic Editor: Ana Paula Arez

Received: 30 August 2023
Revised: 23 September 2023
Accepted: 26 September 2023
Published: 30 September 2023

Copyright: © 2023 by the author. Licensee MDPI, Basel, Switzerland. This article is an open access article distributed under the terms and conditions of the Creative Commons Attribution (CC BY) license (https://creativecommons.org/licenses/by/4.0/).

Supplementary Materials: The following are available online at www.mdpi.com/xxx/s1.

Figure S1: Representation of parasitological and pathological variables (A) number of recovered male worms, (B) number of recovered female worms, (C) eggs per gram of liver and (D) number of granulomas/cm^2 of liver surface between parental strains (CBA/2J, C57/2J) F1 progeny (F1B6BCA) and Backcross (F1BCX). The means and the standard error of the mean (SEM) were represented. Figure S2: Comparison between BX male and female mice: (A) number of recovered male and female worms, (B) number of eggs per gram (EPG) of liver and small intestine, (C) number of granulomas and affected surface of the liver and (D) fecundity of female worms in the liver and small intestine. The means and the standard error of the mean (SEM) were represented. Figure S3: Box plot of BX male and female mice: (A) number of recovered male and female worms; (B) Number of eggs per gram (EPG) of liver and small intestine; (C) number of granulomas and (D) affected liver surface (mm^2/cm^2) of for the BX cohort. Box plot and the mean as a red circle. Figure S4: Dendrogram with all parasitological and pathological variables for schistosomiasis susceptibility: mild in green, mild-moderate in pink, moderate-severe in blue and severe in red. Figure S5: Principal components analysis (PCA). A) Representation of the scores with four tentative groups of mice in the BX cohort. B) Representation of variable loadings. Figure S6: Differences between the four groups of schistosomiasis degree regarding cell lymphocyte subpopulations in splenocytes(A): CD4, CD8, B220 and CD45; cell subpopulations in peripheral blood (B): CD4, CD8, B220; and CD45 and immunoglobulins (C): IgG, Ig1, IgG2a and IgM nine-week postinfection. The comparison among the four groups was made by ANOVA, and the Tukey test was made between every two groups. Figure S7: Syntenic regions located in humans from identified quantitative trace loci (QTL) in mice. A) The QTL1 on mouse chromosome 15 was mainly located on human chromosome 8. B) The QTL2 on mouse chromosome 18 vas mainly located on human chromosome 18. Table S1: Quantitative trace loci (QTL), associated chromosome (Chr) and location (cM) with LDO scores, and the average of the variables in homozygous (AA), heterozygous (AB) and male X alleles (AY and BY) genotypes and the allele combination with higher effect in parasitological and pathological phenotypes in the backcross cohort BX. (EPG- Eggs per gram). Table S2: Quantitative trace loci (QTL), associated chromosome (Chr), location (cM) with LOD scores and the average of the variables in homozygous (AA), heterozygous (AB) and male X alleles (AY and BY) genotypes and the allele combination with higher effect in cell populations at nine-week (W9) post-infection in the backcross cohort BX. Table S3: Quantitative trace loci (QTL), associated chromosome (Chr), location (cM) with LOD scores and average of the variables in homozygous (AA), heterozygous (AB) and male X alleles (AY and BY)

genotypes and the allele combination with higher effect in circulant antibodies at nine-weeks (W9) post-infection in the backcross cohort BX.

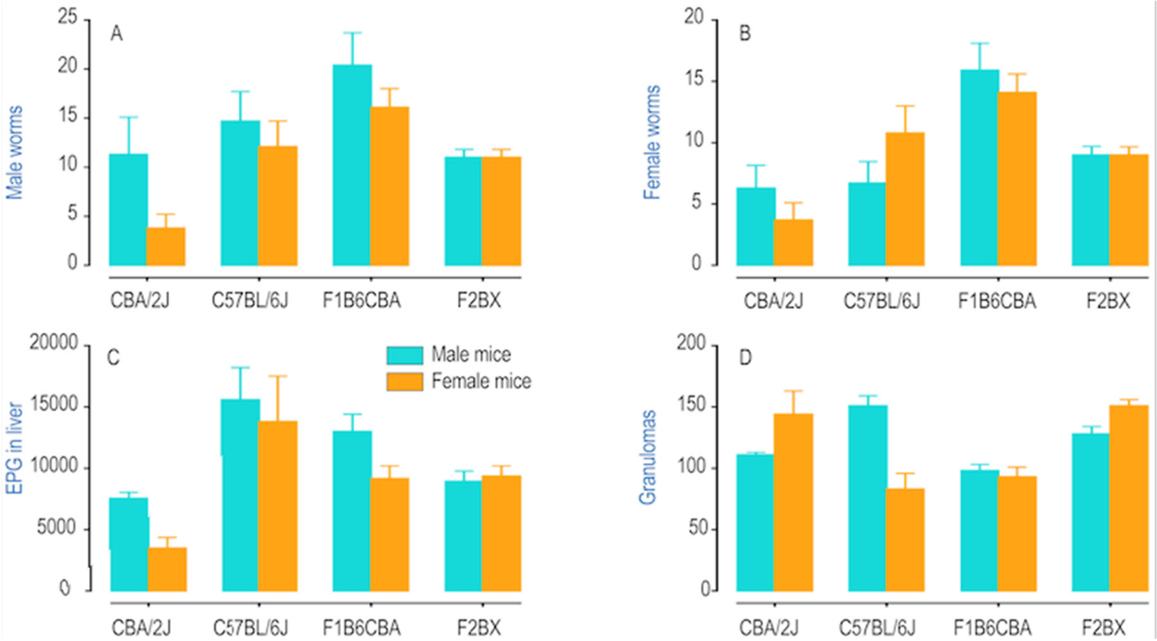

Figure S1. Representation of parasitological and pathological variables (A) number of recovered male worms, (B) number of recovered female worms, (C) eggs per gram of liver and (D) number of granulomas/cm² of liver surface between parental strains (CBA/2J, C57/2J) F1 progeny (F1B6BCA) and Backcross (F1BCX). The means and the standard error of the mean (SEM) were represented.

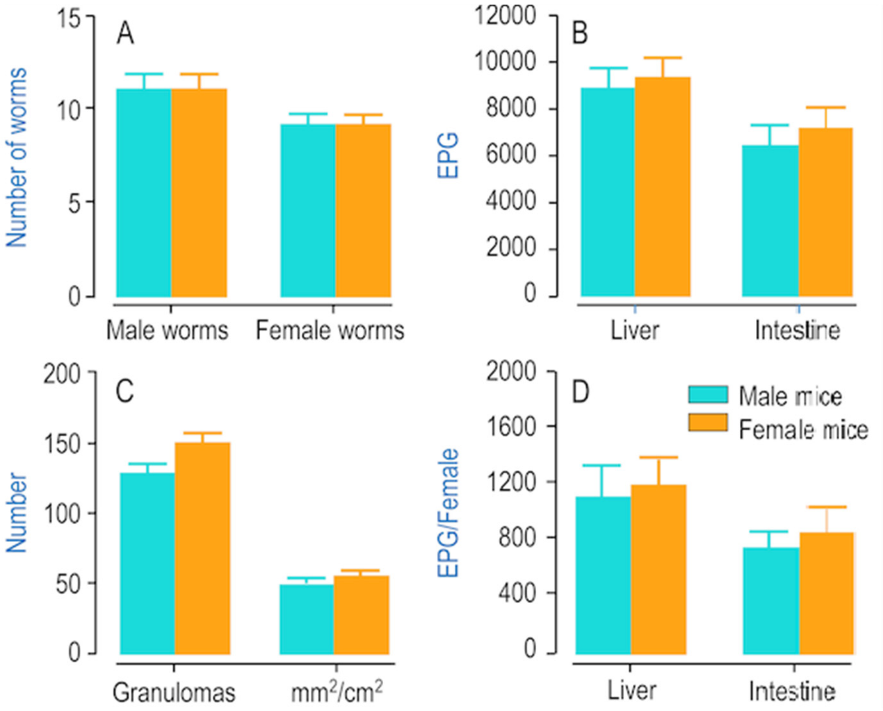

Figure S2. Comparison between BX male and female mice: (A) number of recovered male and female worms, (B) number of eggs per gram (EPG) of liver and small intestine, (C) number of granulomas and affected surface of the liver and (D) fecundity of female worms in liver and small intestine. The means and the standard error of the mean (SEM) were represented.

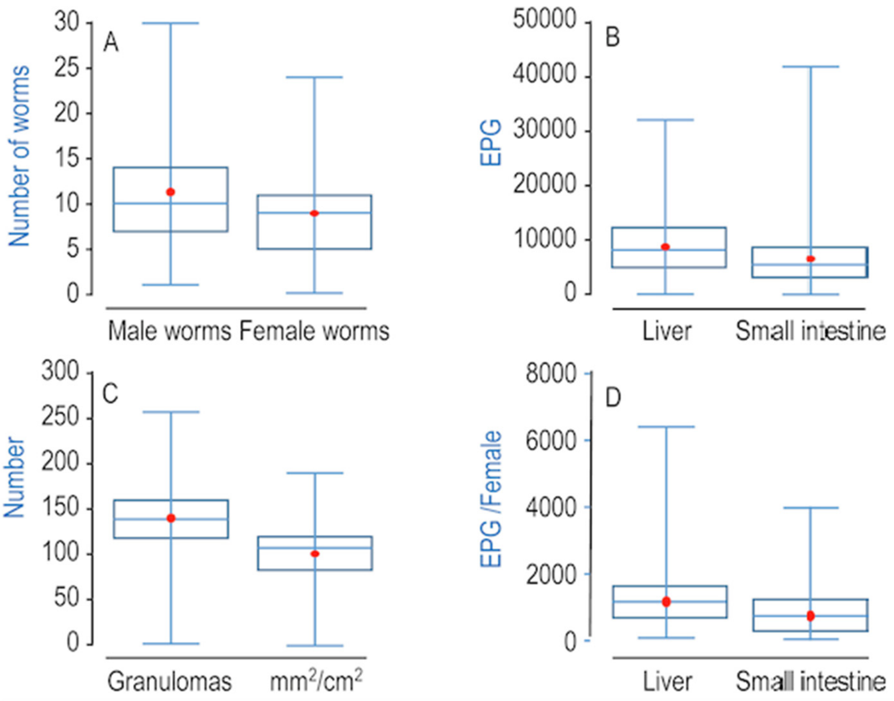

Figure S3. Box plot of BX male and female mice: (A) number of recovered male and female worms; (B) Number of eggs per gram (EPG) of liver and small intestine; (C) number of granulomas and (D) affected liver surface (mm²/cm²) of for the BX cohort. Box plot and the mean as a red circle.

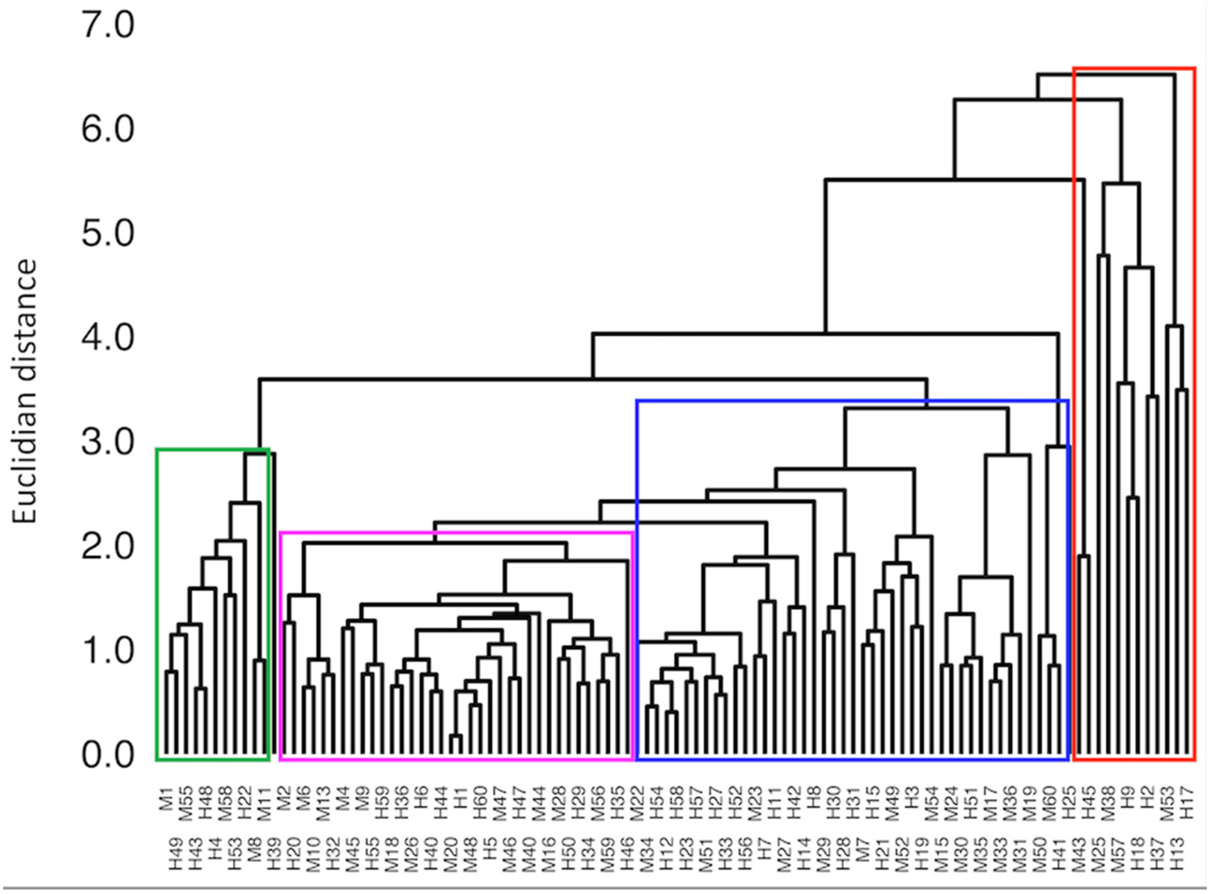

Figure S4. Dendrogram with all parasitological and pathological variables for schistosomiasis susceptibility: mild in green, mild-moderate in pink, moderate-severe in blue and severe in red.

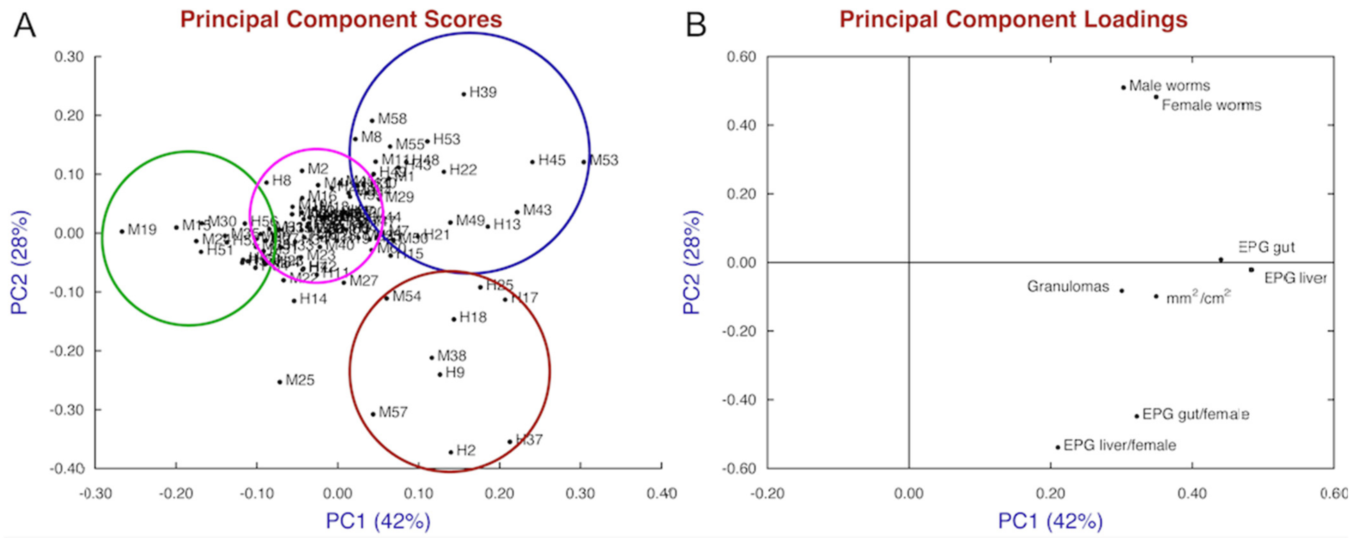

Figure S5. Principal components analysis (PCA). (A) Representation of the scores with four tentative groups of mice in the BX cohort. (B) Representation of variable loadings.

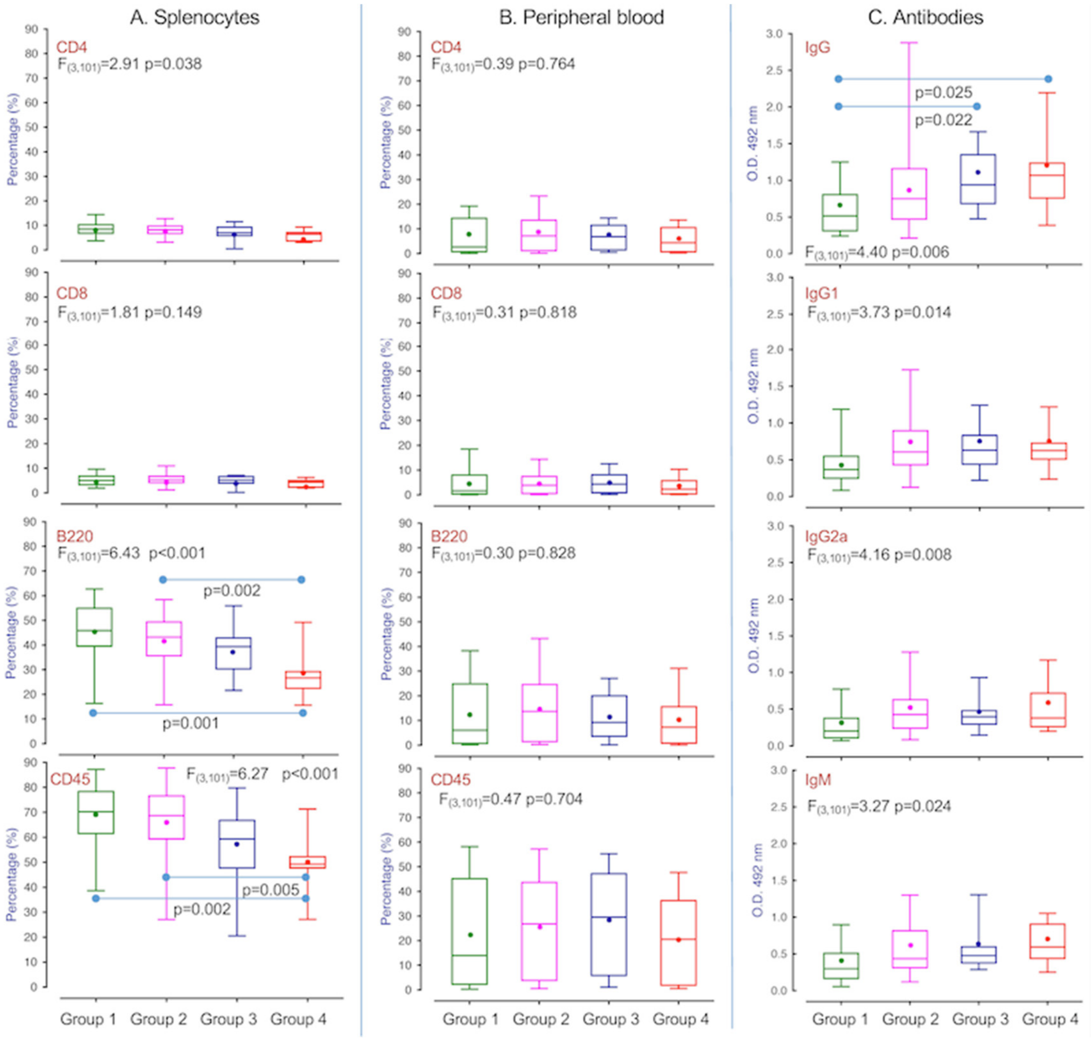

Figure S6. Differences between the four groups of schistosomiasis degree regarding cell lymphocyte subpopulations in splenocytes (A): CD4, CD8, B220 and CD45; cell subpopulations in peripheral blood (B): CD4, CD8, B220 and CD45; and immunoglobulins (C)

IgG, Ig1, IgG2a and IgM nine-week post-infection. The comparison among the four groups was made by ANOVA, and the Tukey test was made between every two groups.

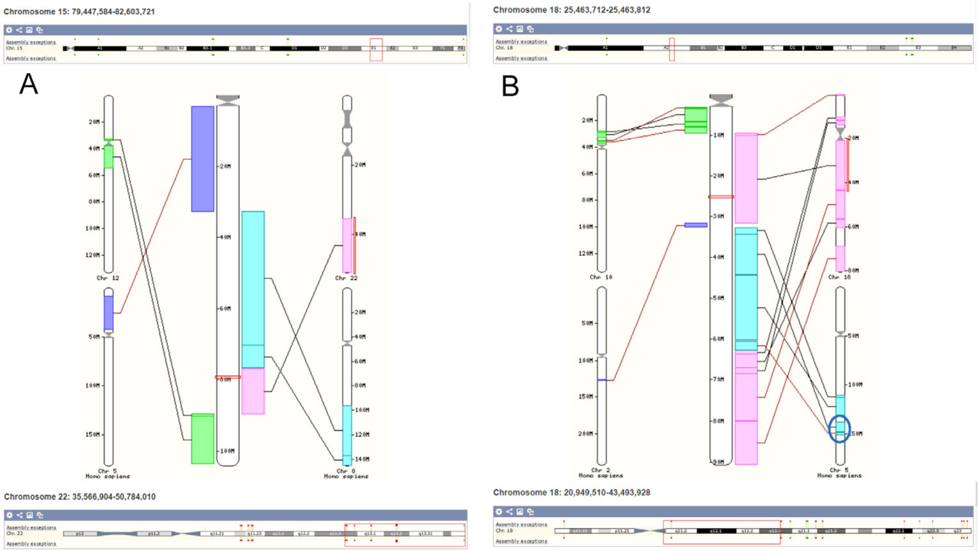

Figure S7. Syntenic regions in human beings from identified quantitative trace loci (QTL) in mice. A) The QTL1 on mouse chromosome 15 was mainly on human chromosome 8. B) The QTL2 on mouse chromosome 18 vas mainly located on human chromosome 18.

Table S1. Quantitative trace loci (QTL), associated chromosome (Chr) and location (cM) with LDO scores, and the average of the variables in homozygous (AA), heterozygous (AB) and male X alleles (AY and BY) genotypes and the allele combination with higher effect in parasitological and pathological phenotypes in the backcross cohort BX. (EPG- Eggs per gram).

Phenotype	eQTL	Chr.	Marker Peak	Location (cM)	LOD Score	Average ± SEM				Higher Effect Allele
						AA	AB	AY	BY	
Granulomas	eQTL1	15	rs6169611	32.17	1.51	152.61±6.49	127.64±6.64			AA
	eQTL2	18	rs13483259	14.18	1.48	129.29±6.40	153.78±6.70			AB
Injured hepatic surface	eQTL3	2	rs13476772	63.25	1.47	56.63±2.76	46.70±2.52			AA
	eQTL1	15	rs13482664	37.72	2.03	56.84±2.56	45.31±2.62			AA
	eQTL4	17	rs13482845	2.52	1.54	45.59±2.80	55.65±2.49			AB
	eQTL2	18	rs13483259	14.18	1.54	46.59±2.55	56.78±2.80			AB
Male worms	eQTL5	6	rs13478799	30.04	2.01	13.11±0.92	9.31±0.80			AA
	eQTL6	7	rs13479382	51.74	1.64	9.31±0.84	12.73±0.88			AB
	eQTL7	X	rs13483769	30.62	2.28	12.81±1.20	10.46±1.08	13.07±1.29	7.74±1.22	AY/AA/AB/BY
Female worms	eQTL5	6	c6.loc57.5	59.38	2.11	10.51±0.73	7.28±0.70			AA
	eQTL7	X	rs13483769	30.62	1.45	9.67±1.03	8.15±0.93	10.73±1.10	7.07±1.05	AY/AA/AB/BY
EPG liver	eQTL8	8	rs13479680	19.01	2.82	6974.25±885.31	11560.76±844.22			AB
	eQTL4	17	rs13474581	4.55	1.41	7481.65±966.22	10806.63±839.62			AB
EPG gut	eQTL9	3	rs13477093	20.78	1.67	5162.24±809.89	8584.90±892.88			AB
	eQTL5	6	rs3023064	7.99	1.50	8424.47±893.53	5169.28±849.21			AA
	eQTL8	8	rs13479680	19.01	1.61	4967.71±869.99	8301.83±831.33			AB
EPG of liver/female	eQTL10	1	rs13475979	47.01	1.49	1685.07±199.20	1031.32±142.09			AA
	eQTL3	2	rs6257221	102.73	1.56	1552.30±159.78	922.42±167.33			AA
	eQTL11	5	rs13478485	60.10	2.37	954.78±142.56	1752.94±185.19			AB
	eQTL12	11	rs13481156	55.29	1.41	967.27±159.11	1573.58±169.49			AB

EPG of gut/female	--	-	-	-	-					

Table S2. Quantitative trace loci (QTL), associated chromosome (Chr), location (cM) with LOD scores and the average of the variables in homozygous (AA), heterozygous (AB) and male X alleles (AY and BY) genotypes and the allele combination with higher effect in cell populations at nine-week (W9) post-infection in the backcross cohort BX.

Phenotype	eQTL	Chr.	Marker Peak	Location (cM)	LOD Score	Average ± SEM				Higher Effect Allele
						AA	AB	AY	BY	
	eQTL13	13	rs13481740	13.23	1.68	5.77±0.83	9.13±0.84			AB
CD4 Blood W9	eQTL14	19	rs13483665	45.23	1.95	9.34±0.86	5.73±0.80			AA
	eQTL15	14	rs8251327	28.08	1.81	7.56±0.73	4.42±0.78			AA
CD8 Blood W9	eQTL4	17	rs13482998	20.63	1.92	7.92±0.80	4.67±0.71			AA
	eQTL13	13	rs13481826	30.69	1.86	30.27±4.21	49.03±4.62			AB
CD45 Blood W9	eQTL15	14	rs8251327	28.08	1.44	46.47±4.61	29.93±4.57			AA
	eQTL14	19	c19.loc40	44.08	1.85	48.68±4.57	30.17±4.26			AA
	eQTL13	13	rs13481826	30.69	1.69	11.65±1.80	19.30±1.98			AB
B220_Blood_W9	eQTL16	19	rs13483665	45.23	1.83	19.42±1.96	11.41±1.81			AA
CD4 Spleen W9	eQTL10	1	rs13475740	4.95	2.03	6.92±0.39	8.56±0.35			AB
	eQTL13	13	rs4229866	35.76	1.68	4.69±.029	5.97±0.33			AB
CD8 Spleen W9	eQTL1	15	rs13482696	43.56	1.49	5.81±0.30	4.65±0.31			AA
CD45 Spleen W9	eQTL10	1	rs13475749	6.03	2.10	58.42±2.34	68.29±1.99			AB
B220 Spleen W9	eQTL4	17	rs13483013	24.72	1.64	42.45±2.56	32.61±2.30			AA

Table S3. Quantitative trace loci (QTL), associated chromosome (Chr), location (cM) with LOD scores and the average of the variables in homozygous (AA), heterozygous (AB) and male X alleles (AY and BY) genotypes and the allele combination with higher effect in circulant antibodies at nine-weeks (W9) post-infection in the backcross cohort BX.

Phenotype	eQTL	Chr.	Marker Peak	Location (cM)	LOD Score	Average ± SEM				Higher Effect Allele
						AA	AB	AY	BY	
	eQTL12	11	rs13481062	38.91	2.03	0.7±0.07	1.02±0.07			AB
IgG W9	eQTL4	17	rs13482845	2.52	3.42	0.15±0.01	0.17±0.009			AB
	eQTL17	16	rs4204623	42.53	2.85	0.48±0.04	0.70±0.04			AB
IgG1 W9	eQTL16	19	rs13483612	32.11	1.74	0.49±0.04	0.67±0.04			AB
	eQTL8	8	rs13479846	37.83	2.13	0.31±0.036	0.48±0.037			AB
IgG2a W9	eQTL18	9	rs4227609	24.58	2.31	0.31±0.035	0.48±0.036			AB
	eQTL19	12	c12.loc57.5	59.92	1.54	0.46±0.037	0.32±0.037			AA
	eQTL7	X	rs13483959	49.16	1.44	0.33±0.047	0.52±0.052	0.34±0.055	0.37±0.053	AB/BY/AY/AA
	eQTL3	2	c2.loc20	23.68	1.57	0.61±0.045	0.44±0.044			AA
IgGM W9	eQTL4	17	rs13482845	2.52	4.96	0.37±0.030	0.67±0.047			AB

Disclaimer/Publisher's Note: The statements, opinions and data contained in all publications are solely those of the individual author(s) and contributor(s) and not of MDPI and/or the editor(s). MDPI and/or the editor(s) disclaim responsibility for any injury to people or property resulting from any ideas, methods, instructions or products referred to in the content.
