# Supplementary figures and images for: Identification of Genomic Regions Implicated in Susceptibility to Schistosoma mansoni Infection in a Murine Backcross Genetic Model
Source: Int J Mol Sci. 2023 Sep 30;24(19):14768. doi: 10.3390/ijms241914768 (PMC10573152; doi:10.3390/ijms241914768)

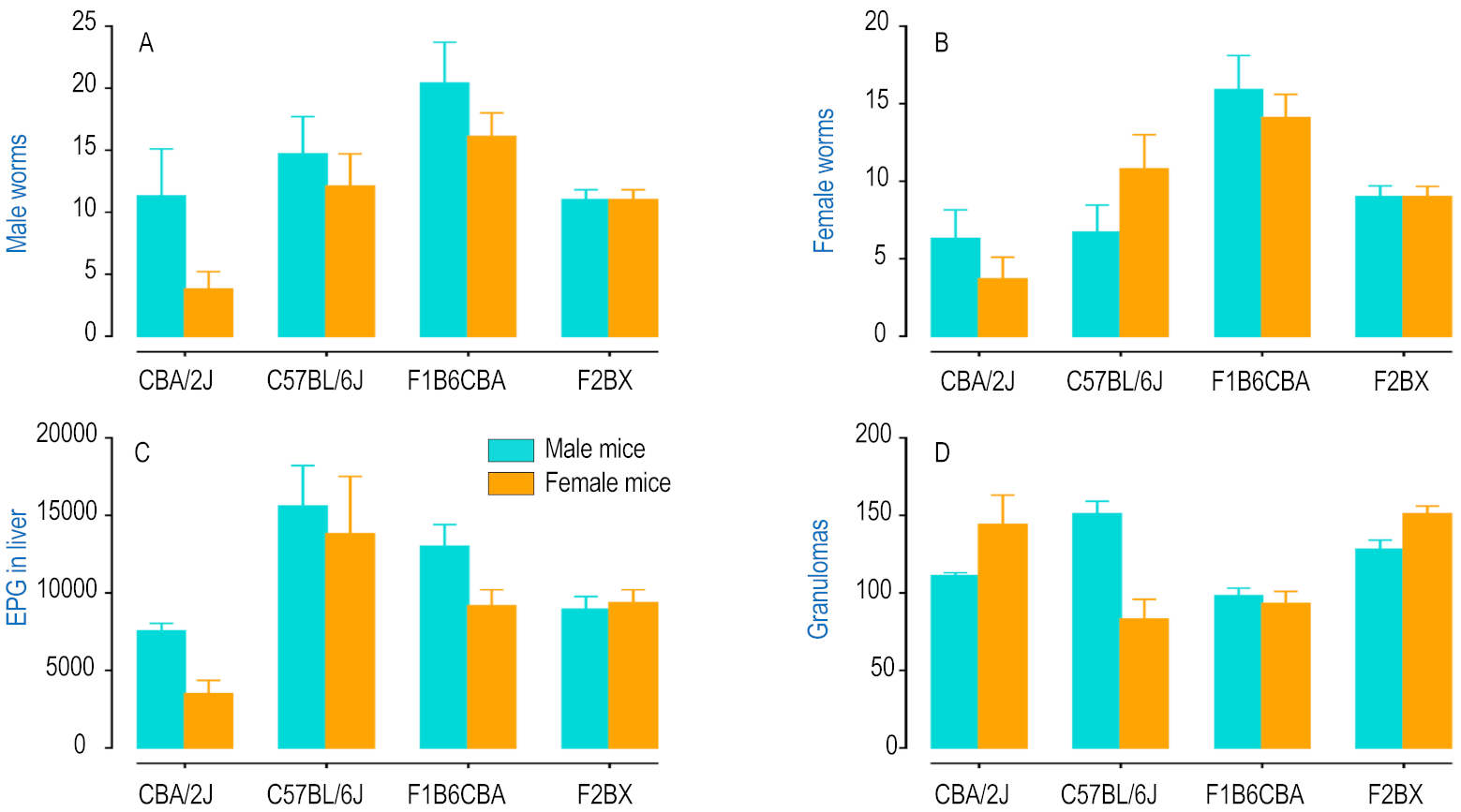

Supplement: Supplementary file 1 [file ijms-24-14768-s001.zip › ijms-2612175 tif Fig S1.tif]

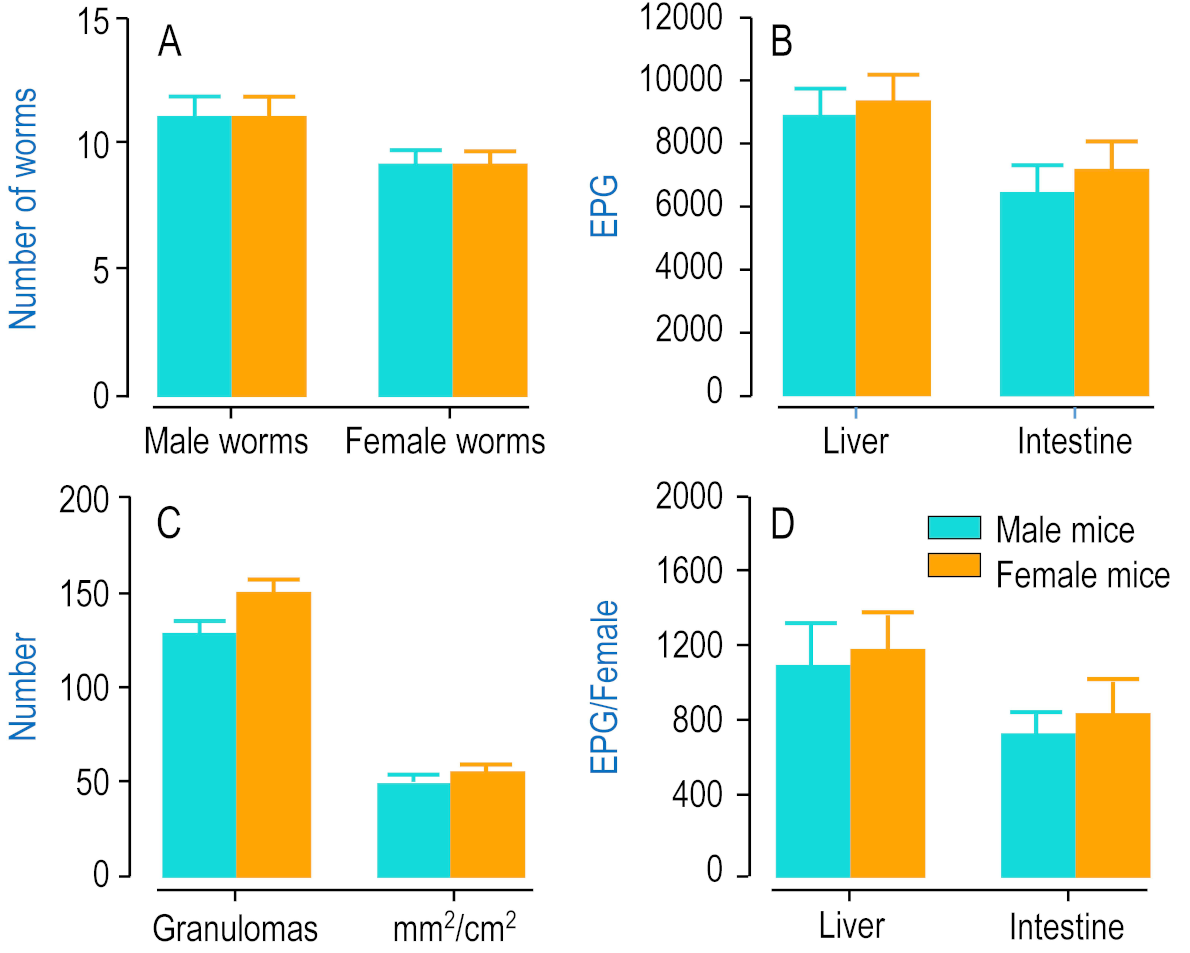

Supplement: Supplementary file 1 [file ijms-24-14768-s001.zip › ijms-2612175 tif Fig S2.tif]

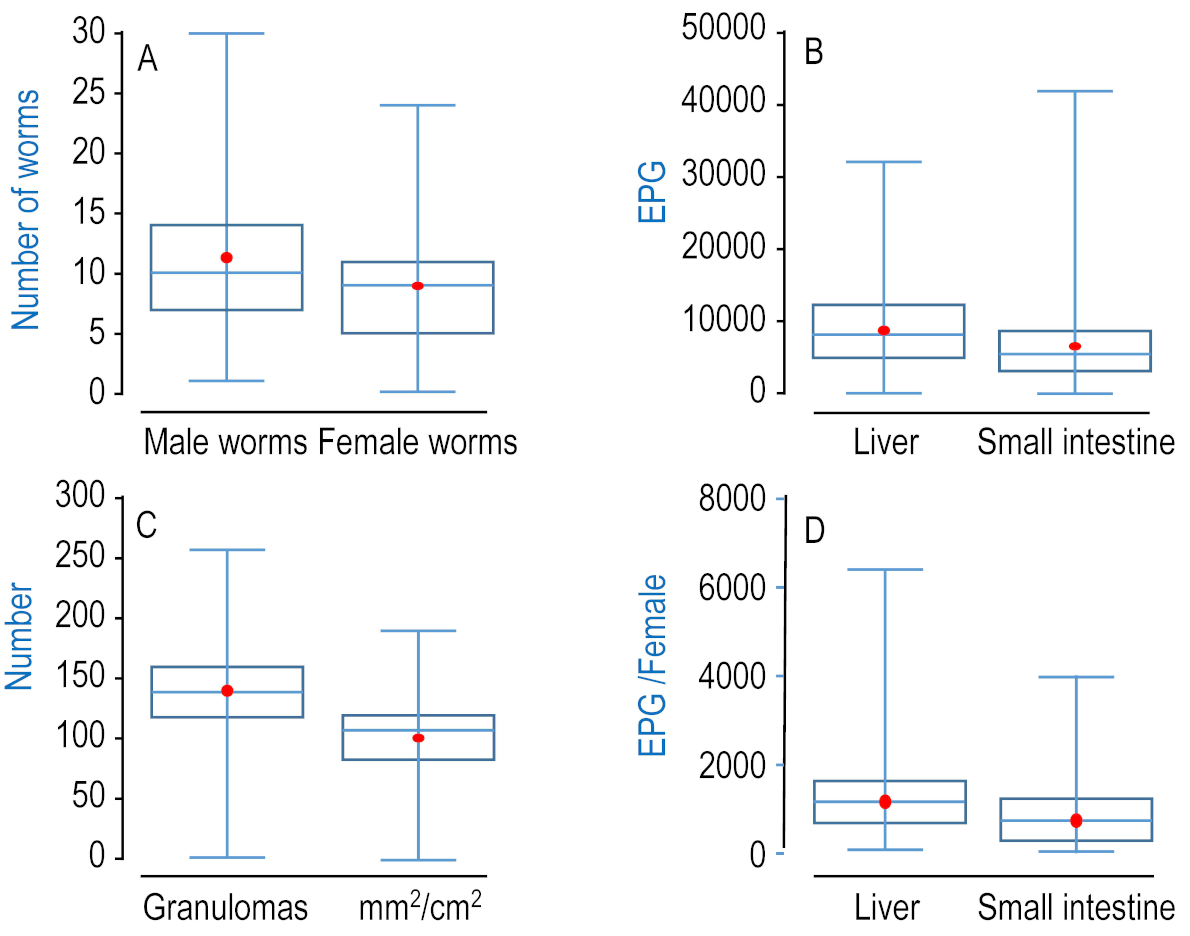

Supplement: Supplementary file 1 [file ijms-24-14768-s001.zip › ijms-2612175 tif Fig S3.tif]

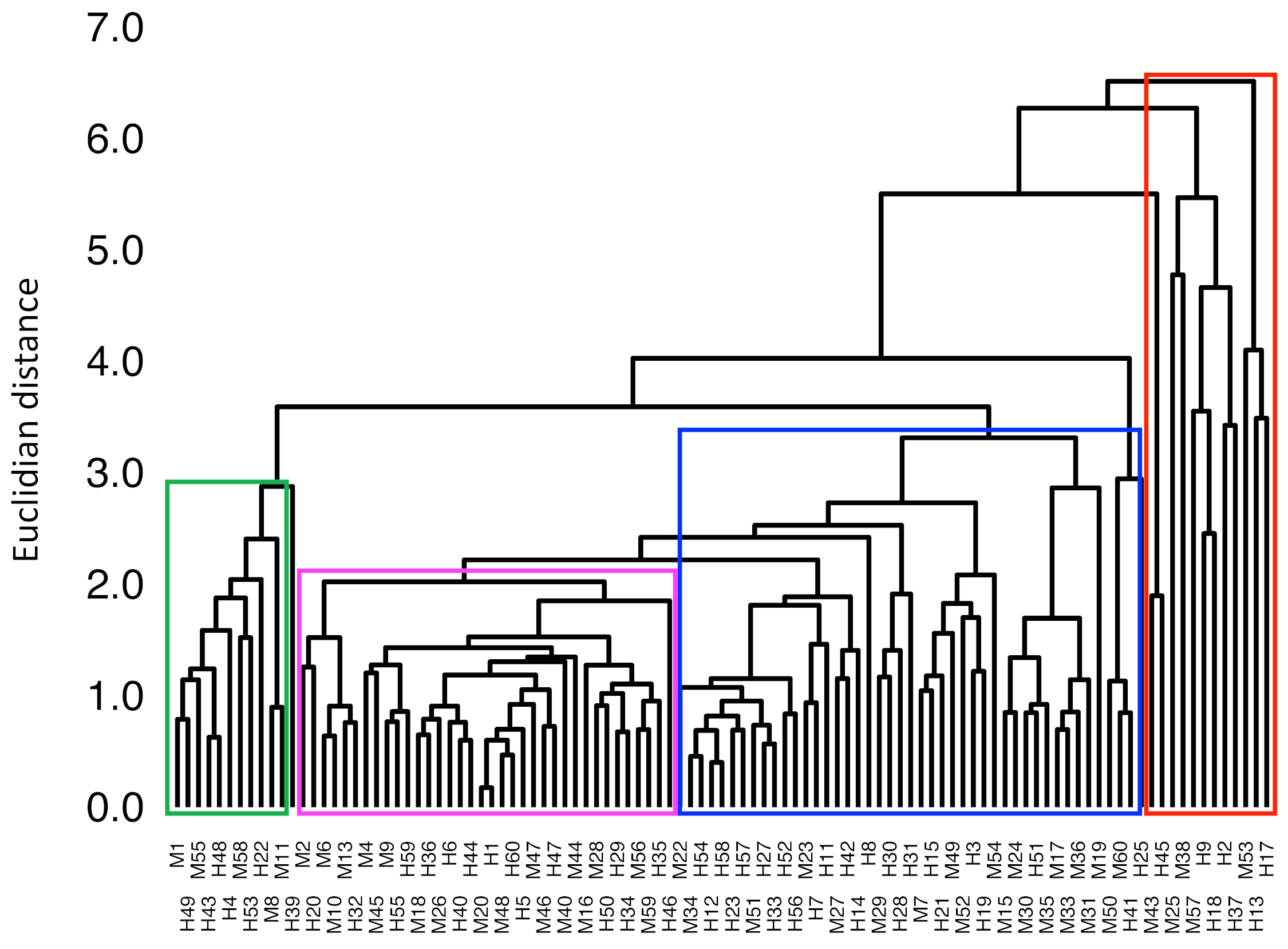

Supplement: Supplementary file 1 [file ijms-24-14768-s001.zip › ijms-2612175 tif Fig S4.tif]

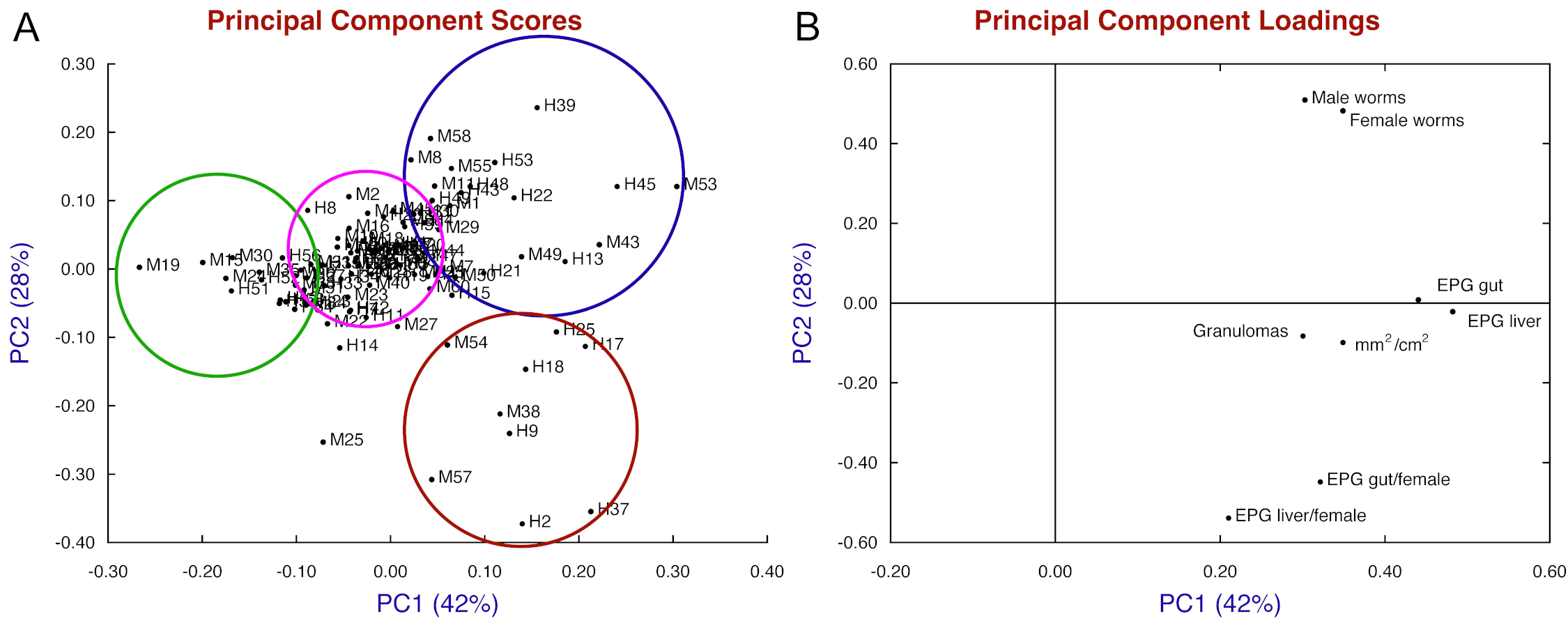

Supplement: Supplementary file 1 [file ijms-24-14768-s001.zip › ijms-2612175 tif Fig S5.tif]

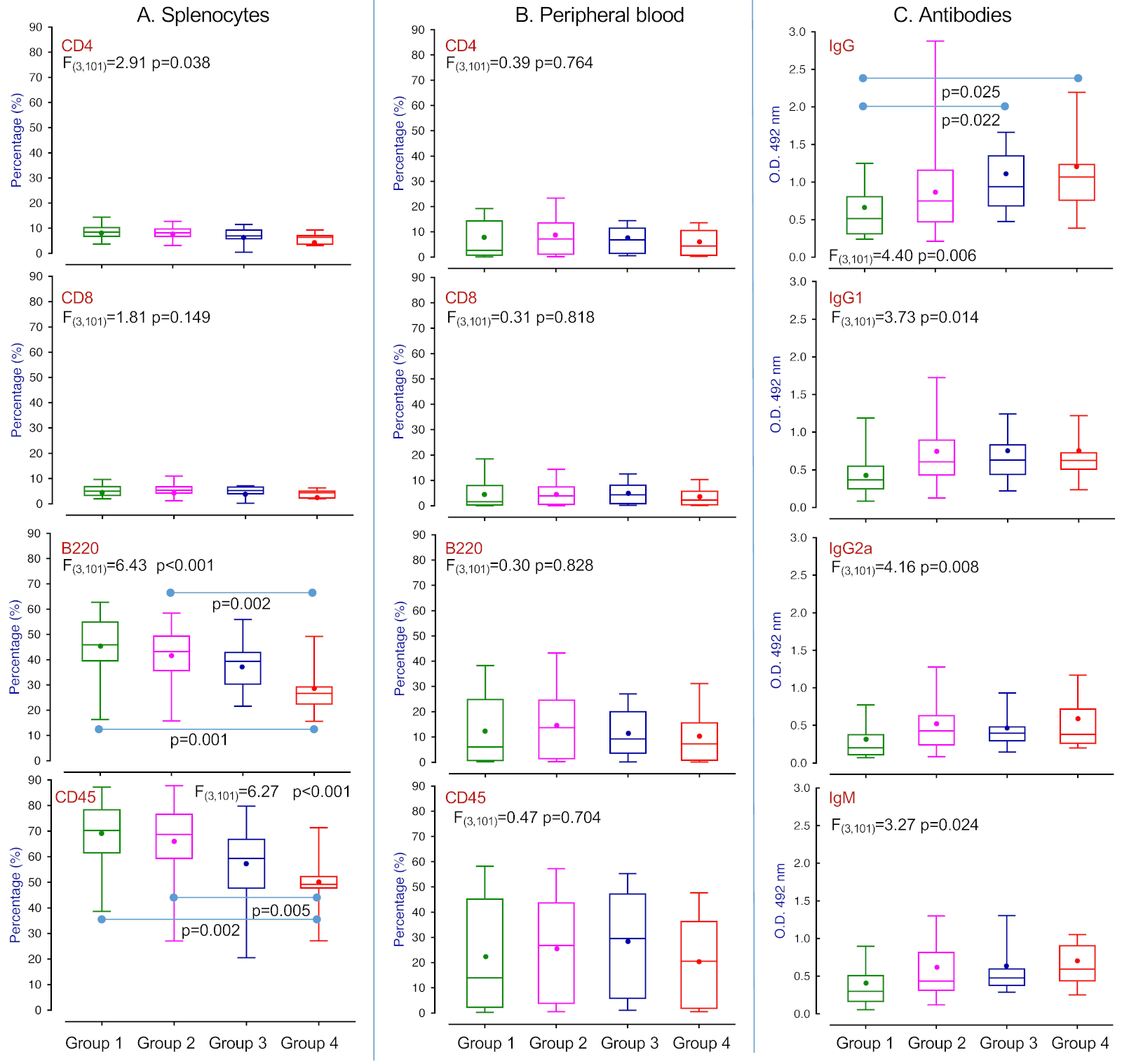

Supplement: Supplementary file 1 [file ijms-24-14768-s001.zip › ijms-2612175 tif Fig S6.tif]

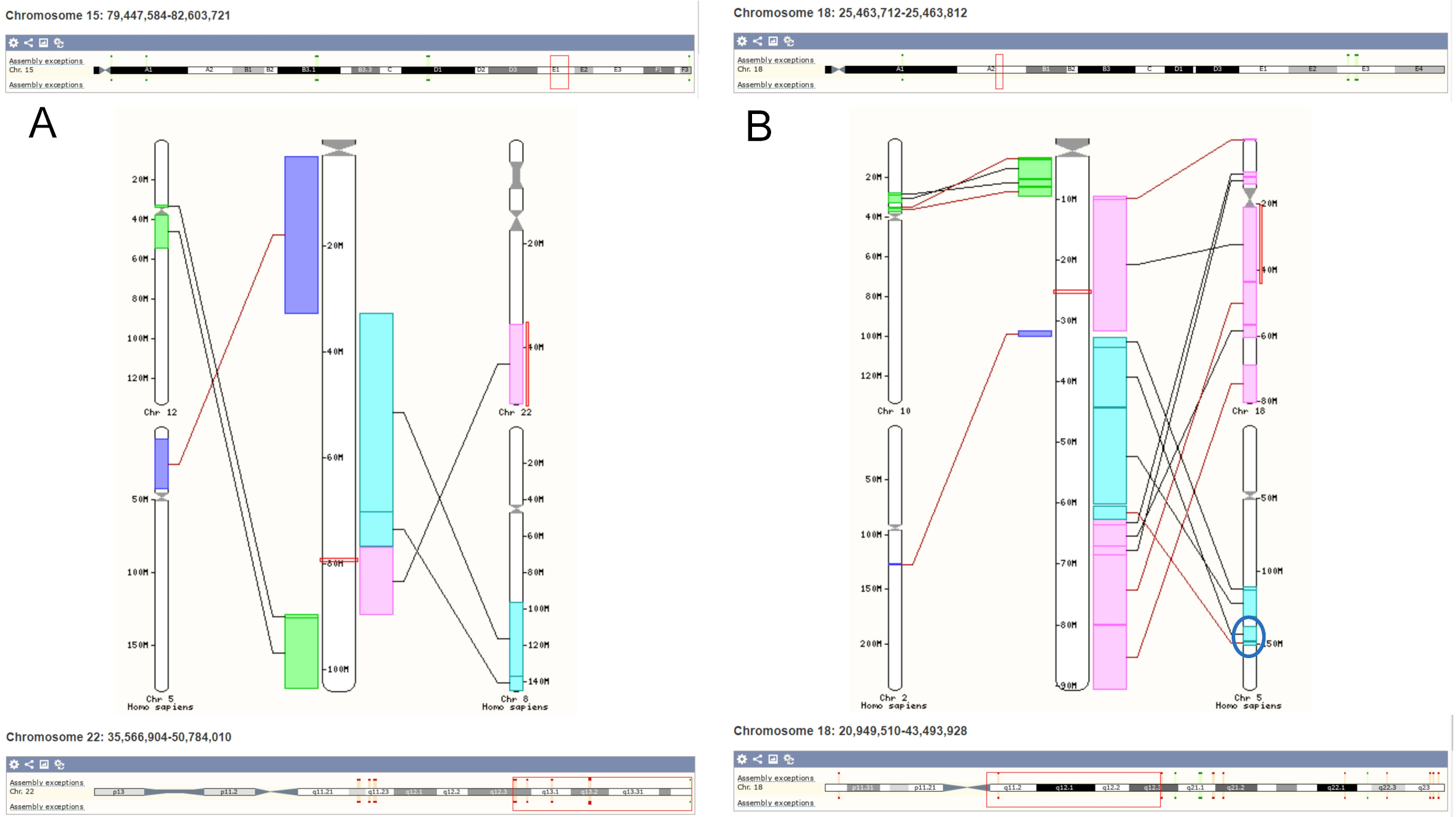

Supplement: Supplementary file 1 [file ijms-24-14768-s001.zip › ijms-2612175 tif Fig S7.tif]
